# Current update on surgical management for spinal tuberculosis: a scientific mapping of worldwide publications

**DOI:** 10.3389/fsurg.2024.1505155

**Published:** 2025-01-24

**Authors:** Romaniyanto Romaniyanto, Muhana Fawwazy Ilyas, Aldebaran Lado, Daffa Sadewa, Dykall Naf'an Dzikri, Enrico Ananda Budiono

**Affiliations:** ^1^ Department of Orthopedic and Traumatology, Prof. Dr. R. Soeharso Orthopedic Hospital, Surakarta, Indonesia; ^2^ Faculty of Medicine, Universitas Sebelas Maret, Surakarta, Indonesia

**Keywords:** bibliometrics, neurosurgery, operative, orthopaedics, scientific mapping, spinal tuberculosis, spinal fusion, surgery

## Abstract

**Introduction:**

Spinal tuberculosis (TB), or Pott's disease, remains a significant global health issue, particularly in regions with high TB prevalence. Despite antitubercular drug therapy being the primary treatment, surgical intervention is often required in cases of spinal instability or neurological complications. This study aims to conduct a comprehensive bibliometric analysis of worldwide publications related to the surgical management of spinal TB and to compare contributions from orthopaedic surgery and neurosurgery in this field.

**Methods:**

A bibliometric analysis was performed using data from the Scopus database, covering publications from 1896 to 2024. The search strategy focused on terms related to spinal TB and surgical interventions. The analysis included 1,857 publications, which were examined for trends, key contributors, and the evolution of surgical techniques. Metrics such as the number of publications, leading authors, affiliations, countries, and funding sponsors were compared between orthopaedic surgery and neurosurgery.

**Results:**

This study identified a steady increase in the number of publications over time. Key topics evolved from basic surgical methods, including early spinal procedures, to integrating pharmacological approaches alongside surgical techniques, such as antitubercular drugs, advancing into imaging research and procedure research involving refined surgical methods like spinal fusion. The recent phase reflects a shift towards technology-driven approaches, including minimally invasive techniques, artificial intelligence, and machine learning. China emerged as the leading country with the most contributions based on author, affiliations, funding sponsors, and countries. Last, orthopaedic surgery had more publications (274) than neurosurgery (96).

**Discussion:**

In conclusion, spinal TB surgery has evolved significantly, with a notable shift towards advanced, technology-driven approaches. Orthopaedic surgery leads in research output compared to neurosurgery. This bibliometric analysis provides valuable insights into the global research landscape, guiding future studies in the management of spinal TB.

## 1 Introduction

Spinal tuberculosis (TB), or Pott's disease, remains a substantial worldwide health concern, especially in areas with elevated TB prevalence, including several developing countries. Globally, TB affects more than 10 million individuals, and annually, around 150,000 new cases of spinal TB are reported (1). India, China, Nigeria, Pakistan, Indonesia, and South Africa comprise 64 percent of all documented cases (2). The incidence rate of TB increased by 3.6% in 2021 compared to 2020 (3). Subsequently, the burden of drug-resistant TB rose by 3% from 2020 to 2021, with 450,000 recorded cases of rifampicin-resistant TB in 2021. Russia and other nations in Eastern Europe and Central Asia documented the most significant percentages (>50%) of multidrug-resistant or rifampicin-resistant TB among persons previously treated (3). In addition, spinal TB represents <1% of all TB cases (4, 5). This disease can also lead to debilitating consequences, including severe deformities, neurological deficits, and even paraplegia if not managed appropriately (6). While antitubercular drug therapy is the cornerstone of treatment, its limitations become evident in advanced cases, particularly when there is deformity, spinal instability or neurological complications (7, 8). The incidence of neurological complications varies between 10% and 43% (4). Medical therapy alone may be insufficient in such scenarios, necessitating surgical intervention (9, 10).

Surgical management, particularly within the orthopaedic and neurosurgery disciplines, plays a crucial role in managing spinal TB, especially when conservative treatment fails (10). Although the roles of each discipline can overlap, surgery is often required to stabilize the spine, decompress neural elements, prevent or correct deformities, alleviate pain, and improve neurological outcomes (11–14). Over the years, advancements in surgical techniques have been reported to be significantly beneficial for patients with spinal TB. Several meta-analyses have been conducted to compare each approach. However, the results are varied because of several factors, including the location of the disease, the patient's comorbidities, the extent of spinal involvement, and others. Additionally, approach consideration is also essential; some studies favour the posterior approach (15–17), some anterior approach (18), and some both the posterior-only approach and combined posterior-anterior approach (19). In addition, recently, several minimal invasive approaches have also been reported, for instance, using video-assisted thoracic surgery (VATS) (20–24), minimally invasive pedicle screw fixation (25–27), and minimally invasive far lateral debridement combined with posterior instrumentation (MI-FLDPI) (28).

Bibliometric analysis, which quantitatively studies scientific publications, offers valuable insights into research trends, key contributors, and emerging areas of interest (29–31). Bibliometric analysis has been performed in the scope of medicine and health, which may provide valuable insights into research trends, key contributors, and emerging areas, aiding in the evolution of medical practices and guiding future research and policy decisions (32, 33). By examining the global research landscape, bibliometric studies can elucidate the evolution of surgical management to spinal TB, guiding future research and informing policy decisions. Therefore, this study aims to perform a comprehensive bibliometric and scientific mapping analysis of worldwide publications on surgical interventions in managing spinal TB. A comparison of the contributions of orthopaedics and neurosurgery in this field is also performed.

## 2 Methods

### 2.1 Data sources and search strategy

A comprehensive bibliometric analysis was conducted for scientific mapping following procedures from previous studies (34–42). The data was obtained from the Scopus database online. Before selecting Scopus as the primary database, it was compared with other databases, including PubMed and ScienceDirect, to evaluate coverage and relevance. Then, Scopus was chosen because it provides the most comprehensive metadata compatible with Biblioshiny software and includes more relevant publications on spinal TB than other databases. Subsequently, to mitigate the bias introduced by daily database changes, the search process was executed on Saturday, August 31, 2024. Keywords utilized in this study were (“spinal tuberculosis” OR “spinal TB” OR “tuberculous spondylitis” OR “pott's disease” OR “pott disease”) AND (surger* OR surgeo* OR surgical OR operative). The preliminary search identified 2,886 studies. Only literature published in English that had attained the final publication stage was included. Subsequently, the document types included are articles, reviews, conference papers, and book chapters. Then, all the studies that met our requirements based on the title and abstract were screened, and any irrelevant studies were eliminated. Finally, 1,857 studies were included in this study. Additionally, to compare orthopaedics and neurosurgery publications on this topic, the keywords (“spinal tuberculosis” OR “spinal TB” OR “tuberculous spondylitis” OR “pott's disease” OR “pott disease”) AND (orthopedi* OR orthopaedi*) were used for orthopaedics publication searching. After screening, we revealed a total of 274 publications included. Subsequently, (“spinal tuberculosis” OR “spinal TB” OR “tuberculous spondylitis” OR “pott's disease” OR “pott disease”) AND (neurosurge*) were used for neurosurgery publication searching, and a total of 96 publications were included after the screening process.

### 2.2 Data analysis

The publication output was analyzed using Scopus analysis tools, R package's bibliometrics (Biblioshiny) (43–45), and VOSviewer (version 1.6.18) (46, 47). Scopus analysis tools were used to obtain the top ten most relevant authors, affiliations, countries, funding sponsors, and the most influential publications. The R package's bibliometrics (Biblioshiny) utility was designed for quantitative scientometrics and informetrics. This study used Biblioshiny to describe included studies, annual scientific production, its impacts, and trend topics. In trend topics, the visualization was set for four topics per year. Subsequently, a bibliometric network of keywords co-occurrence for prominent or key topics was analyzed using VOSviewer. In this study, the visualization of prominent or key topics was set on a minimum occurrence of seven and a minimum total link of the strength of 5 using VOSviewer. Inconsistent or evolving terminology over time was addressed by standardizing keywords, replacing synonymous terms, unifying terminology with different spellings, and grouping keywords with similar meanings.

## 3 Results

### 3.1 Description of included studies

This study included 1,857 publications published from 1896 to 2024, sourced from 621 different journals, books, and other academic publications. The average annual growth rate of publications is 3.24%. Each document has an average of 17.67 citations, and there are 33,354 references cited across all documents. The dataset includes 6,659 Keywords Plus terms and 2,441 author-defined keywords. Subsequently, there are 5,699 authors, with 159 single-authored documents and an average of 4.7 co-authors per document. International co-authorship accounts for 5.708% of the total. Last, document types are predominantly research articles (1,599), with additional contributions from 42 book chapters, 48 conference papers, and 168 review articles.

### 3.2 Annual scientific production and impacts

Over the years, the number of published articles in the field has fluctuated significantly. Early periods such as 1896 and 1920 saw only a single article each year with very few citations, reflecting limited research output. However, the volume of publications increased substantially from the mid-20th century onwards, peaking in 2022 with 148 articles. Despite this increase in the number of articles, the mean total citations per article has shown considerable variation. In some years, such as 2006, the average number of citations per article was notably high at 31.68, indicating a period of significant impact and recognition. Conversely, in recent years like 2022, the average number of citations per article decreased to 1.87, suggesting a shift in citation patterns and possibly an increase in the number of articles with fewer citations or due to their recent publication, which means they have had less time to accumulate citations compared to older articles.

**Figure 1:**
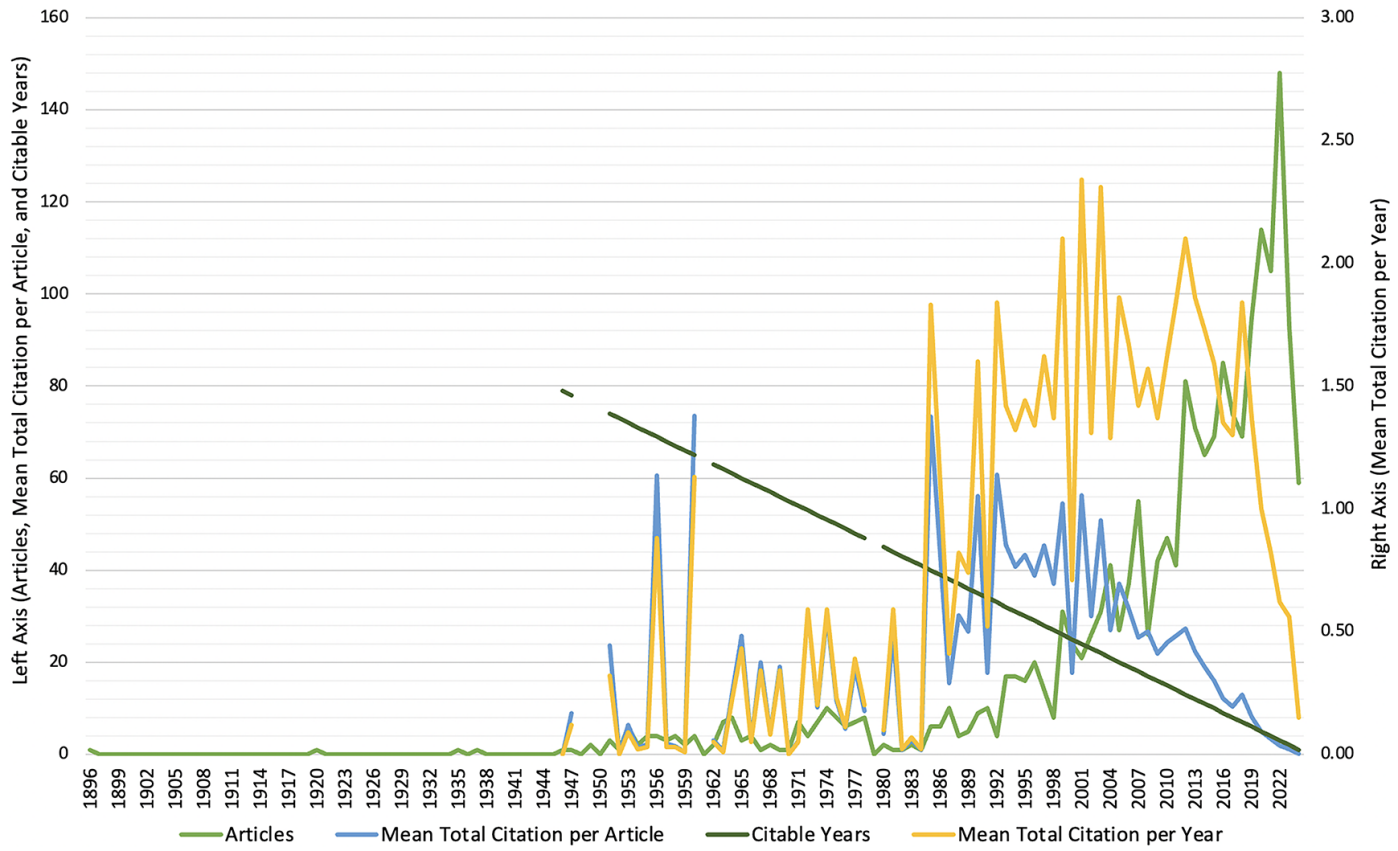
Annual scientific production and impacts on the role of surgery on spinal tuberculosis.

The mean total citations per year reflects the average number of citations received by articles published in a specific year. This metric has exhibited notable peaks, such as in 1990, when articles had an average of 56.11 citations per year, indicating strong citation impact. Similar to previous metrics, this figure has declined recently, with 2022 showing an average of only 1.87 citations per year. Subsequently, citable years measures how long articles have been available for citation and provides context for understanding citation longevity. Older articles from the early 20th century have had more time to accumulate citations, while more recent articles have had less time to be cited. This metric highlights articles’ ongoing relevance and potential impact over time, influencing the interpretation of citation data and research trends. All of the details of annual scientific production and its impacts are visualized in Figure 1.

### 3.3 Most relevant authors, affiliations, countries, and funding sponsors

**Figure 2:**
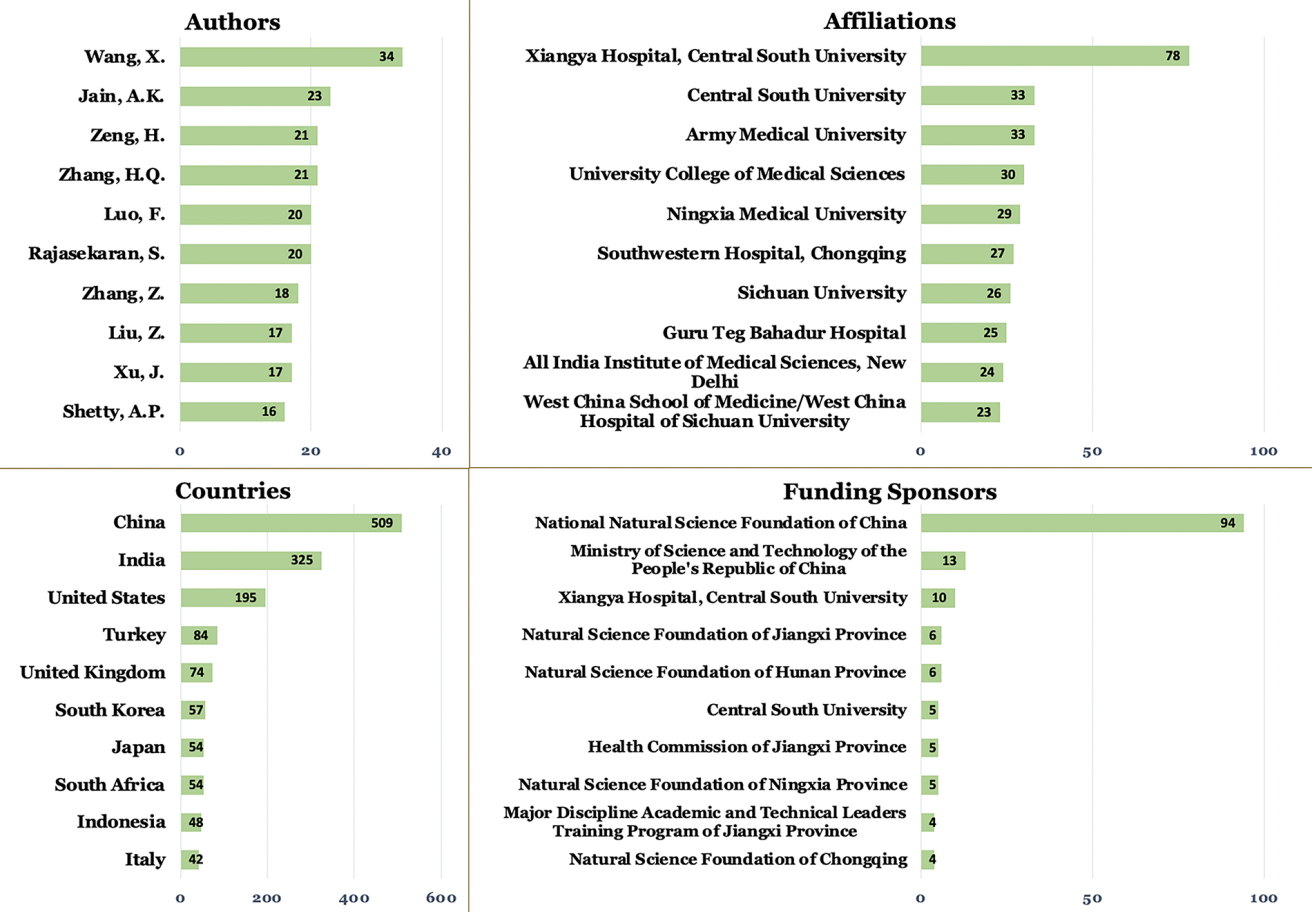
Most relevant authors, affiliations, countries, and funding sponsors on the role of surgery on spinal tuberculosis.

Most relevant authors, affiliations, countries, and funding sponsors on surgical management for spinal TB are visualized in Figure 2. Wang, X. (34 publications), Jain, A.K. (23 publications), and Zeng, H. (21 publications) as the most prolific authors in the field. Subsequently, the leading affiliations include Xiangya Hospital Central South University (78 publications) and Central South University and Army Medical University, each with 33 publications. In addition, China emerged as the most prominent country, accounting for 509 publications, followed by India with 325 and the United States with 195. Last, the National Natural Science Foundation of China (94 publications) was the top funding sponsor, followed by the Ministry of Science and Technology of the People's Republic of China (13 publications) and Xiangya Hospital, Central South University (10 publications).

### 3.4 Most influential publications

Among the entire publications in the field of surgery roles on spinal TB, the article “Spinal tuberculosis: A review,” published in the Journal of Spinal Cord Medicine in 2011, stands out with a total of 460 citations and an average of 35.38 citations per year. Another key publication is “Tuberculosis of the spine: A fresh look at an old disease,” which appeared in the Journal of Bone and Joint Surgery in 2010, with 325 citations and an average of 23.21 citations per year. The article “Anterior spinal fusion. The operative approach and pathological findings in 412 patients with Pott's disease of the spine,” published in The British Journal of Surgery, published in 1960, is also highly influential. It has garnered 292 citations, averaging 4.56 citations per year. The ten most influential publications on the role of surgery on spinal TB are displayed in Table 1.

**Table 1:** Most influential publications on the role of surgery on spinal tuberculosis.

Rank	Title	Journal	Year	Total citation	Total citations per year
1	Spinal tuberculosis: A review (48)	Journal of Spinal Cord Medicine	2011	460	35.38
2	Tuberculosis of the spine: A fresh look at an old disease (49)	Journal of Bone and Joint Surgery	2010	325	23.21
3	Anterior spinal fusion. The operative approach and pathological findings in 412 patients with Pott's disease of the spine (50)	The British Journal of Surgery	1960	292	4.56
4	Spine update tuberculosis of the spine: Controversies and a new challenge (51)	Spine	1997	273	10.11
5	Spinal tuberculosis in adults: A study of 103 cases in a developed country, 1980–1994 (52)	Medicine	1999	265	10.6
6	Spinal tuberculosis (Pott's disease): Its clinical presentation, surgical management, and outcome. A survey study on 694 patients (5)	Neurosurgical Review	2001	262	11.39
7	Tuberculosis of the central nervous system (53)	Postgraduate Medical Journal	1999	252	10.08
8	Spinal tuberculosis: A diagnostic and management challenge (54)	Journal of Neurosugery	1995	242	8.34
9	Anterior spinal fusion a preliminary communication on the radical treatment of pott's disease and pott's paraplegia (55)	British Journal of Surgery	1956	242	3.56
10	Evaluation of the risk of instrumentation as a foreign body in spinal tuberculosis: Clinical and biologic study (56)	Spine	1993	228	7.35

### 3.5 Trend topics

This scientific mapping finds that the evolution of spinal surgery management for treating spinal TB publications has progressed from a basic foundation in early surgical methods, the integration of pharmacological approaches alongside surgical techniques, advancing into imaging research, procedure studies with refined surgical methods, and most recently, a technology-driven phase incorporating innovations like artificial intelligence and machine learning. The basic foundation was laid in the 1950s and 1960s when the focus was on early surgical methods, which was evident from terms like “tuberculosis, spinal/therapy” and “surgical procedures, operative.” Subsequently, as the field progressed, there was a significant integration of pharmacological approaches alongside surgical techniques. Terms like “aminosalicylic acid,” “isoniazid,” “rifampin,” “ethambutol,” and “pyrazinamide,” and “drug therapy” highlight the emergence of combining medications with surgery to improve patient outcomes.

**Figure 3:**
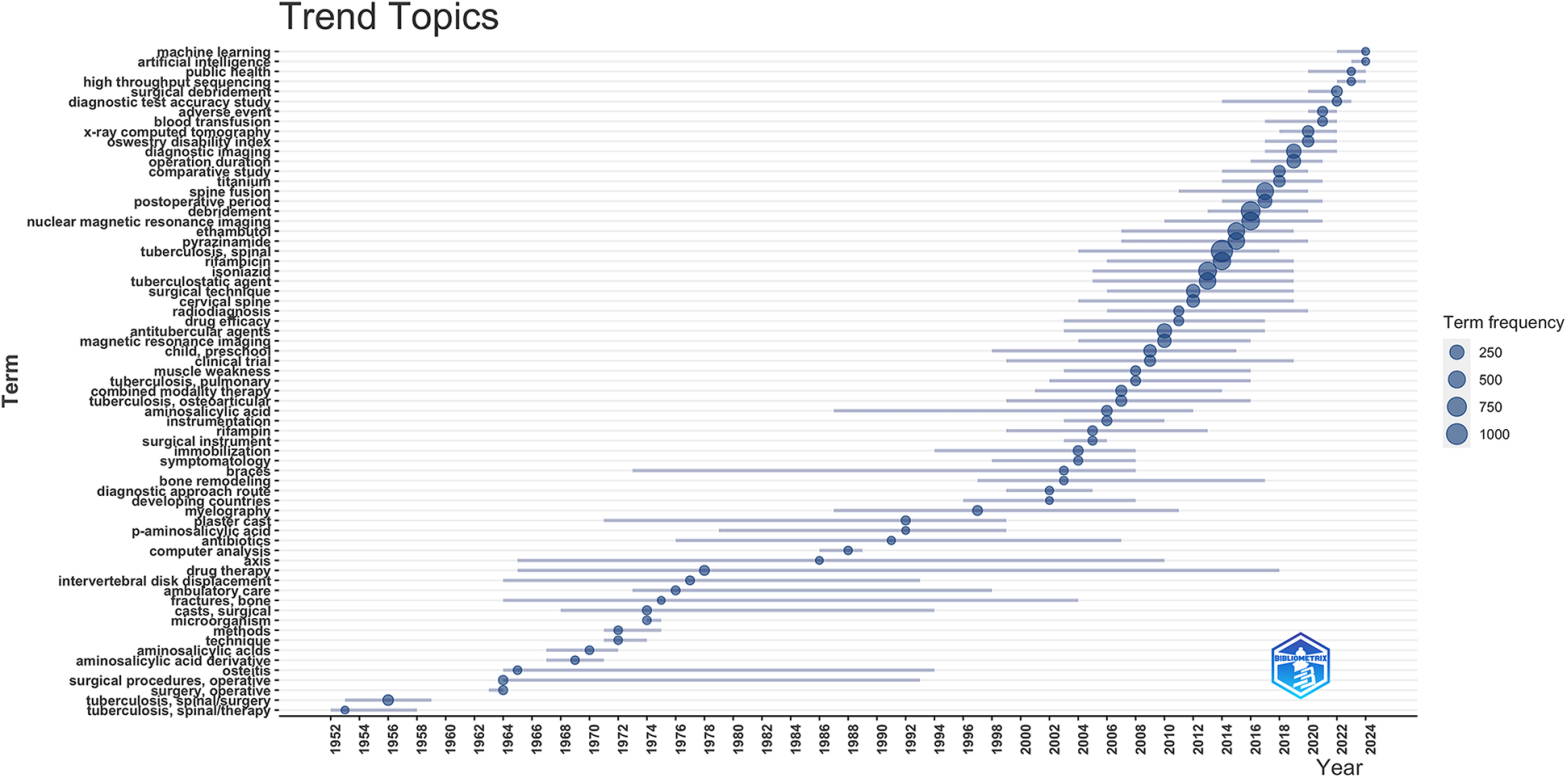
Trend topics on the role of surgery on spinal tuberculosis.

The subsequent period marked a shift toward imaging research and procedure research, with increased attention on diagnostic tools like “magnetic resonance imaging (MRI)” and “myelography.” In addition, advancements in “surgical instrumentation” and the development of complex procedures, including “spine fusion,” reflected the ongoing refinement of surgical methods. The field has entered a technology-driven phase in recent years, incorporating cutting-edge tools like “artificial intelligence” and “machine learning.” This latest trend indicates a move towards highly specialized, technology-enhanced approaches in managing spinal TB, reflecting the continuous evolution of the field. The entire trend topics on the role of surgery on spinal TB are visualized in Figure 3.

### 3.6 Prominent/key and underdeveloped topics

**Figure 4:**
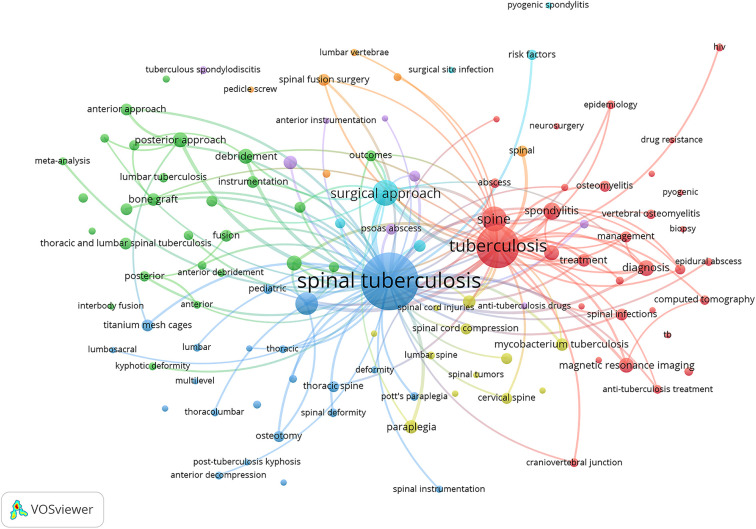
Prominent/key topics on the role of surgery on spinal tuberculosis.

In the context of surgery for spinal TB, there are more common keywords, for instance posterior approach (with occurrence of 38), minimally invasive spine surgery (30), bone graft (27), anterior approach (25), instrumentation (25), posterior approach only (24), spinal fusion surgery (24), titanium mesh cages (24), chemotherapy (20), anti-tuberculosis treatment (14), combined anterior and posterior approach (14), posterior instrumentation (14), meta-analysis (12), anterior debridement (10), anterior decompression (10), and anterior instrumentation (8). These more common keywords, which represent prominent/key topics on the role of surgery on spinal TB, are visualized in Figure 4. We set only keywords with a minimum occurrence of 7 that are visualized in that Figure, and the line between keywords was set on the minimum of 5 strength.

Subsequently, less common keywords, which may suggest emerging or underexplored areas, include arthrodesis (3), machine learning (3), short-segment fixation (3), video-assisted thoracoscopic surgery (3), artificial intelligence (2), biportal endoscopic spine surgery (2), circumferential decompression (2), circumferential fusion (2), closing-opening wedge (2), intraoperative neurophysiological monitoring (2), lateral extracavitary approach (2), oswestry disability index (2), pedicle screw fixation (2), pedicle screw instrumentation (2), pedicle subtraction osteotomy (2), polymethylmethacrylate (2), posterior transforaminal lumbar debridement (2), posterolateral approaches (2), posterolateral decompression (2), retroperitoneal approach (2), revision surgery (2), single-segment fixation (2), thoracolumbar corpectomy (2), transforaminal lumbar interbody fusion (TLIF) (2), transoral surgery (2), transpedicular approach (2), transpedicular corpectomy (2), transpedicular instrumentation (2), two-stage surgery (2), unilateral limited laminectomy (2), and video-assisted thoracic surgery (2).

### 3.7 Comparison of orthopedics surgery and neurosurgery publications

In comparing the metrics between orthopaedic surgery and neurosurgery in the context of spinal TB, orthopaedic surgery stands out with a higher total number of publications, totalling 274, compared to 96 in neurosurgery. The leading author in orthopaedic surgery is Wang, X. with ten publications, while Kumar, R. is the most prominent in neurosurgery with three publications. Xiangya Hospital Central South University leads in orthopaedic surgery affiliations with 14 publications, whereas Sanjay Gandhi Postgraduate Institute of Medical Sciences tops neurosurgery with four publications. China is the top country contributing to orthopaedic surgery, with 85 publications, while the United States leads neurosurgery with 20 publications. The National Natural Science Foundation of China is the top funding sponsor for orthopaedic surgery, supporting 18 publications. In neurosurgery, the Education Department of Jiangxi Province and others support two publications. Last, the Spine journal is the leading publication outlet for orthopaedic surgery with 18 articles, whereas World Neurosurgery is the top journal in neurosurgery with 13 articles. Entire comparison metrics can be seen in Table 2.

**Table 2:** Comparison of orthopedics surgery and neurosurgery publications on the role of surgery on spinal tuberculosis.

Metrics	Orthopedics surgery	Neurosurgery
Total publications	274 publications	96 publications
Top Authors	Wang, X. (10) Liu, Z. (5) Luo, F. (5)	Kumar, R. (3) Srivastava, A.K. (3) Ali, M. (2)
Top Affiliations	Xiangya Hospital Central South University (14) Army Medical University (10) Southwestern Hospital Chongqing (8)	Sanjay Gandhi Postgraduate Institute of Medical Sciences (4) University of Cape Town (2) Groote Schuur Hospital (2)
Top countries	China (85) United States (37) India (33)	United States (20) China (14) India (12)
Top funding sponsors	National Natural Science Foundation of China (18) Ministry of Science and Technology of the People's Republic of China (4) Science and Technology Department of Sichuan Province (2)	Education Department of Jiangxi Province (2) Medical Research Council (2) National Natural Science Foundation of China (2)
Top Journals	Spine (18) International Orthopaedics (13) Clinical Orthopaedics and Related Research (10)	World Neurosurgery (13) Journal Of Neurosurgery (5) Turkish Neurosurgery (3)

## 4 Discussion

### 4.1 Overview of findings

This study provides a comprehensive bibliometric analysis of the global research landscape concerning the surgical management of spinal TB, spanning publications from 1896 to 2024. The analysis revealed a steady increase in scholarly output, with a significant surge in the number of publications in recent decades. This growth reflects the increasing recognition of spinal TB as a critical health issue and the evolving role of surgery in its management. Orthopaedic surgery has contributed more to this field than neurosurgery, as evidenced by more publications.

Orthopaedic surgery often focuses on structural stability, deformity correction, and spinal reconstruction, which are critical in addressing the primary manifestations of spinal TB. Conversely, neurosurgery's contributions, though fewer in volume, remain indispensable, particularly in refining surgical strategies to address neurological deficits and optimize outcomes for cases with severe spinal cord involvement. This distinction highlights the complementary expertise of the two specialities and reinforces the importance of multidisciplinary collaboration between orthopaedic surgeons and neurosurgeons. This comparison also holds important global health implications. Spinal TB is a significant burden in low-resource settings, and understanding the dominant and non-dominant contributors can help policymakers and researchers allocate efforts to bridge gaps. These efforts might include increasing neurosurgical training or resources, especially in underserved regions.

China has emerged as a dominant contributor to research in this field, with leading authors, institutions, and funding agencies driving much of the scholarly work. In the entire work, the role of surgery in spinal TB publications from China has gained 5,595 citations and 35 h-index. The reasons behind China's high publication output are possibly due to a multifaceted phenomenon driven by several interconnected factors. First, China faces a significant TB burden. The World Health Organization indicates that China is the second highest among the 22 countries with the most significant TB burden (57), possibly making TB a critical priority. Subsequently, China's centralized healthcare system facilitates large-scale data collection and analysis, and the implementation of integrated TB control models in primary healthcare sectors has enabled comprehensive studies on TB control effectiveness (58, 59). In addition, recent reforms in China's research funding landscape have aimed to improve efficiency and support promising scholars. For instance, the NSFC has implemented measures to curtail personal favours in the review process, reform scoring mechanisms for distinguished young scholars, and initiate funding programs for outstanding doctoral and undergraduate students (60). These policy changes may create a more competitive and productive research environment, potentially contributing to increased publication output.

Additionally, China has been actively fostering international collaborations in medical research. The analysis of collaborative networks between institutions and countries reveals a global “North-South” connection between developed and developing nations (61, 62). These partnerships may not only enhance the quality of research but also increase the likelihood of publications in high-impact international journals. Lastly, applying big data resources in medical research has significantly boosted China's research capabilities. Integrating big data in medical collaborative networks has improved the transaction efficiency of medical services, enabling more sophisticated and comprehensive studies. It has likely contributed to the quantity and quality of publications (63).

Subsequently, the evolution of key topics over time highlights the progression of surgical techniques and the integration of multidisciplinary approaches. Initially, research focused on the basic foundation of surgical methods, such as early spinal procedures and the use of antitubercular drugs. The first publication in this scope in the Scopus database was a case report entitled “Pott's disease. Its surgical treatment, with a report of a case” authored by Punton, J. and published in the Journal of Nervous and Mental Disease in 1896 (64). Over time, the focus shifted toward more advanced procedures, incorporating imaging research and refined surgical techniques like spinal fusion. In recent years, the field has moved towards technology-driven approaches, emphasizing minimally invasive techniques, the incorporation of artificial intelligence, and machine learning to improve surgical outcomes and patient care.

These findings are relatively the same as those of a previous study that describes the timeframe for spine surgery for TB over the last seven decades, which has progressed through the pre-chemotherapy, biological, mechanical, and technological phases (65). Mandar et al. described that most studies discussed debridement and extirpation of the diseased tissue approach in the pre-chemotherapeutic era. Subsequently, the biological era focused on anterior debridement and non-instrumented fusion approaches. In addition, debridement with instrumented (anterior/posterior) fusion has been discussed in the mechanical era. Last, the recent technological era describes minimally invasive techniques, thoracoscopic techniques, local drug delivery systems, and bioactive materials (65).

### 4.2 Main topics

In this study, the posterior approach emerged as the most frequently discussed surgical technique in treating spinal TB, with 38 occurrences. Closely related is the “posterior approach only” method, which was mentioned 24 times. In comparison, the anterior approach was only mentioned 25 times, and the combined anterior and posterior approach was cited 14 times. More studies on posterior approaches were possible because of the higher prevalence of thoracolumbar spinal TB cases that recommended using the posterior approach.

Several high-level evidence studies, including systematic reviews and meta-analyses, have been discussed about these approaches and their combination. Liu et al. study stated that the posterior approach demonstrated equivalent clinical efficacy while reducing operation time, blood loss, hospital stay, and complications compared to the combined posterior and anterior approach in managing spinal TB (15). Subsequently, another meta-analysis by Muheremu et al. showed that the correction of the Cobb angle by the posterior approach is considerably greater than that achieved through the anterior approach (16). Another study, including comparing anterior, posterior, and combined approaches, concluded that the posterior technique yields superior clinical outcomes compared to the anterior or combination approaches for spinal TB (17). Another study showed a different result, as the anterior approach has been reported to present fewer complications than both the combined and posterior-only approaches (18). Last, a different study showed that both approaches can yield excellent clinical results. The posterior-only approach can reliably and efficiently accomplish lesion debridement, decompression, and the reconstruction and maintenance of stability, offering benefits including reduced invasiveness, diminished bleeding, shorter operative duration and hospital stay, and fewer complications, appearing to surpass the combined posterior-anterior approach (19).

In general, the choice of surgical approach could depend on several factors, including lesion location, extent of bone destruction, deformity correction needs, patient's condition, or surgeon's expertise. The location of the lesion is essential; for instance, cervicothoracic spinal TB may require either a single-stage anterior approach or a combined anterior-posterior approach, depending on the relation between the TB lesion segments and the suprasternal notch (67). Severe destruction of the anterior column may need an anterior or combined approach (68). Subsequently, the posterior-only and combined anterior-posterior approaches have shown higher correction rates for kyphotic deformity than the anterior-only approach (68). In addition, the patient's overall health also matters, as the posterior approach involves shorter surgery and less blood loss, making it a potentially better option for those in poor condition (68, 69). Last, the surgeon's experience with different techniques also plays a significant role in deciding the approach.

The analysis also highlighted the growing focus on advanced surgical techniques, particularly minimally invasive spine surgery (30 occurrences). This approach has gained traction due to its potential for reduced recovery time, decreased postoperative complications, and improved patient outcomes. Alongside these techniques, instrumentation, which was mentioned 25 times and titanium mesh cages (24 occurrences) underscores the advancements in surgical tools that have enhanced the effectiveness of spinal fusion surgeries. Subsequently, bone grafting, mentioned 27 times, remains a cornerstone technique in spinal TB surgery, particularly in reconstructing spinal stability following debridement. These supportive techniques are essential for addressing the structural damage caused by spinal TB, preventing further deformities, and enhancing the patient's quality of life post-surgery.

Additionally, the initial two decades of the 21st century (2001–2020), termed the technological era, featured the advent of diverse treatments, including less invasive techniques for treating spinal TB (70–75). Subsequently, instrumentation has shown that it plays a crucial role in managing spinal TB by providing mechanical stability and enhancing the healing process (65, 76). Various surgical techniques involving instrumentation include, for instance, posterior decompression and fusion with bone autografts, anterior debridement/decompression and fusion with bone autografts, and posterior fusion with instrumentation followed by simultaneous or sequential anterior debridement/decompression and fusion (9). In addition, previous meta-analyses (77, 78), which include several studies (79–89), have been studied about titanium mesh cages and bone grafting. Those studies concluded that both bone grafting and titanium mesh grafting are both effective and safe surgical procedures, with no significant statistical differences in outcomes.

Alongside surgical interventions, the use of chemotherapy (20 occurrences) or anti-tuberculosis treatment (14 occurrences) continues to be a critical component of managing spinal TB. A previous study has discussed the beneficial effect of chemotherapy for spinal TB, especially in mild spinal TB (90), as well as there is no indication for surgery (6). Subsequently, previous meta-analyses (91, 92), which included several studies (93–100), have been discussed to compare the duration of chemotherapy and its effect. Those studies show that short-course chemotherapy (≤6 months) is as effective as standard chemotherapy (≥nine months) in treating spinal TB. Last, emerging techniques such as anterior debridement (10 occurrences), anterior decompression (10 occurrences), and anterior instrumentation (8 occurrences) indicate a growing interest in refining and expanding the surgical toolkit available to clinicians (65).

### 4.3 Research gaps and future directions

This study showed several studies need to be conducted in the future. First, even minimally invasive approaches have been studied and included in the main topics; there are no systematic reviews and meta-analyses in this scope. Future research should prioritize such studies to provide high-quality evidence on the comparative benefits of these techniques. Subsequently, areas including the utilization of artificial intelligence, machine learning, and robotics have also emerged recently and potentially can be future directions of studies. A previous study stated that recently, modern spine surgery is a multi-disciplinary endeavour involving not only the surgeon but also possibly artificial intelligence and robotic technologies (65). A study showed that using Artificial Intelligence in the form of computer navigation-assisted minimally invasive direct lateral interbody fusion may substantially decrease intraoperative radiation exposure without extending overall operation duration (101). Subsequently, machine learning could facilitate the prediction of extended hospital stays post-surgery and discover risk factors for tuberculous spondylitis patients with imbalanced data, employing a unique approach utilizing explainable artificial intelligence (XAI). That study employs an XGBoost model, readily accessible via the deployed web application, which can facilitate clinical research (102). Another study employing MVITV2, EfficientNet-B3, ResNet101, and ResNet34 as backbone networks and developing deep learning models demonstrated that sagittal images based on T2WI can effectively differentiate between spinal TB and spinal metastases (SM), achieving diagnostic performance comparable to that of experienced spine surgeons (103). Last, there is a study on developing and validating an innovative predictive model and web-based calculator assessing transfusion risk following spinal fusion for spinal TB (104).

Regarding the role of robotics technology, which has also emerged recently, The awarded Surgical Team of the Year was represented by Ravinder Uberoi from Apollo Hospitals in New Delhi. The researchers successfully performed robotic spinal surgery on a ten-year-old kid suffering from bone destruction due to spinal TB, which they characterised as a “world's first” (105). Subsequently, a study about treating thoracolumbar TB using robot-assisted and minimally invasive techniques through a transforaminal expansion method compared with traditional posterior open surgery, robot-assisted minimally invasive access through the transforaminal approach for lesion removal and bone grafting internal fixation in thoracolumbar TB treatment can decrease the operative duration and intraoperative haemorrhage, lessen surgical trauma, and achieve definitive effectiveness (106). Additionally, a study about the accuracy and safety of pedicle screw placement assisted by orthopaedic robot and C-arm fluoroscopy, including 1 case of spinal TB, showed orthopaedic robot-assisted pedicle screw placement offers advantages such as reduced fluoroscopy duration, decreased screw placement time, and enhanced precision, hence augmenting surgical safety and presenting significant potential for application in orthopaedics (107).

Thoracic pedicle screw insertion assisted by the TiRobot system for spinal TB also demonstrates favourable outcomes and holds potential for further analysis (108). In this case, they present the use of robot-assisted navigation in a complex post-tubercular pediatric kyphotic deformity. Subsequently, robotic technology coupled with navigation and integrated intraoperative CT scans could also allow precise instrumentation, reduced complications, lower radiation exposure, and better patient outcomes, especially in complex deformity cases (109). Last, there is a meta-analysis that included 7,379 pedicle screws showed that robotic-assisted surgery demonstrated significantly higher perfect pedicle screw accuracy compared to the freehand technique and also lower complication rates, proximal-facet joint violation, radiation exposure, although not specific to the spinal TB cases (110). Future studies should focus on refining these technologies, exploring their cost-effectiveness, and assessing their scalability, particularly in resource-limited settings where the burden of TB is highest.

Collectively, these advancements enhance the safety, efficiency, and effectiveness of spinal TB surgeries, marking a significant step forward in managing this challenging condition. However, the adoption of these advanced technologies may face several challenges. First, high costs may limit accessibility, particularly in low-resource settings where TB is most prevalent. Secondly, specialized training is required for surgeons and staff to use these tools effectively. Moreover, integrating artificial intelligence and robotics into already overburdened healthcare systems may pose logistical challenges. Last, ethical and legal concerns, such as patient data privacy and liability in artificial intelligence-driven decision-making, may further complicate widespread adoption. Addressing these issues is essential for maximizing the clinical potential of these technologies in spinal TB management. Therefore, future research should also focus on improving access to advanced surgical technologies and specialized care in low-resource settings. This could help address global inequalities in spinal TB management by developing scalable, cost-effective solutions that bridge the gap between high-income and low-resource healthcare systems.

An additional avenue for future research could also involve integrating advanced techniques and materials for managing spinal TB inspired by innovations in related or other fields. For instance, the Neuroendoscopic Parafascicular Evacuation of Spontaneous Intracerebral Hemorrhage (NESICH) technique, which has demonstrated utility in minimizing brain tissue damage and providing effective access for evacuating hematomas (111), may serve as an example model for minimally invasive approaches in spinal infections that may potentially reduce collateral tissue damage during surgical interventions. Similarly, image-guided techniques, such as those employed in corticospinal tract approaches for hematoma evacuation, have been shown to enhance surgical precision and reduce complications in managing delicate neural structures (112). These methods could also be adapted for guiding surgical approaches around the spinal cord in spinal TB, where precision is critical for preserving neurological function. Moreover, integrating regenerative approaches, such as the use of apoptotic bodies derived from 3D-cultured adipose stem cells, which have shown promise in enhancing tissue repair and promoting healing in ischemic flap studies (113), can also be studied for potential in accelerating post-surgical recovery in spinal TB by facilitating tissue regeneration. Last, nanotechnology may also offer a promising frontier, with nanomaterials already proving effective in bone metastasis treatment (114). These materials could be explored further for their applications in spinal TB to improve drug delivery, enhance infection control, and support bone tissue regeneration.

## 5 Limitations

Despite the comprehensive nature of this bibliometric analysis, several limitations must be acknowledged. First, the study relied solely on the Scopus database for data retrieval, which, although extensive, may not capture all relevant publications. Second, the analysis focused primarily on quantitative metrics such as the number of publications, citations, and author affiliations, which, while informative, may not fully capture the qualitative aspects of the research, such as the clinical impact or real-world applicability of the findings. However, to address this, the discussion section incorporates qualitative insights summarizing key advancements, such as minimally invasive techniques and the integration of artificial intelligence, to provide context and relevance to the findings. Additionally, the reliance on publication and citation counts as an indicator of influence or importance, which may introduce a bias towards more recent works, potentially overlooking less cited but still significant contributions to the field. Another limitation is categorizing publications into orthopaedic and neurosurgical domains based on explicit mentions, which may not fully capture the multidisciplinary nature of spinal TB treatment, where collaboration between orthopaedic surgeons, neurosurgeons, and other specialists is common. Future studies are necessary to explore and document interdisciplinary practices better, especially in employing qualitative methods, such as expert interviews or case reviews.

## 6 Conclusion

This bibliometric analysis of worldwide publications on surgical management for spinal TB reveals a significant evolution in the field, marked by advancements in surgical techniques and the increasing integration of technology. Over the years, there has been a steady increase in research output, reflecting the growing recognition of the importance of surgical intervention in managing spinal TB, particularly in cases of spinal instability or neurological complications. Subsequently, the comparison between orthopaedic surgery and neurosurgery publications reveals that orthopaedic surgery has a higher volume of research output in this field, indicating its predominant role in spinal TB management. However, both disciplines contribute valuable insights and advancements to the surgical treatment of this challenging condition.

The study highlights key trends in the evolution of surgical methods, from basic early techniques to the adoption of minimally invasive procedures and the recent incorporation of artificial intelligence and machine learning into surgical practice. The posterior approach remains the most frequently discussed surgical method, but there is also significant interest in anterior approaches, minimally invasive techniques, and the use of advanced instrumentation. The combination of surgical intervention with chemotherapy and anti-TB treatment further underscores the importance of a multidisciplinary approach to managing spinal TB. This study provides a detailed overview of the global research landscape on surgical management for spinal TB, offering valuable guidance for future research and clinical practice development in managing this debilitating disease.

## Data Availability

The raw data supporting the conclusions of this article will be made available by the authors, without undue reservation.

## References

[1] Pawar UM Kundnani V Agashe V Nene A Nene A. Multidrug-resistant tuberculosis of the spine—is it the beginning of the end? Spine (Phila Pa 1976). (2009) 34:E806–10. 10.1097/BRS.0b013e3181af7797 19829244

[2] Shanmuganathan R Ramachandran K Shetty AP Kanna RM. Active tuberculosis of spine: current updates. N Am Spine Soc J. (2023) 16:100267. 10.1016/j.xnsj.2023.100267 37736557 PMC10510092

[3] Bagcchi S. WHO’s global tuberculosis report 2022. Lancet Microbe. (2023) 4:e20. 10.1016/S2666-5247(22)00359-7 36521512

[4] Kiran NAS Vaishya S Kale SS Sharma BS Mahapatra AK. Surgical results in patients with tuberculosis of the spine and severe lower-extremity motor deficits: a retrospective study of 48 patients. J Neurosurg Spine. (2007) 6:320–6. 10.3171/spi.2007.6.4.6 17436920

[5] Turgut M. Spinal tuberculosis (Pott’s disease): its clinical presentation, surgical management, and outcome. A survey study on 694 patients. Neurosurg Rev. (2001) 24:8–13. 10.1007/PL00011973 11339471

[6] Rajasekaran S Soundararajan DCR Shetty AP Kanna RM. Spinal tuberculosis: current concepts. Global Spine J. (2018) 8:96S–108. 10.1177/2192568218769053 30574444 PMC6295815

[7] Vasakova M. Challenges of antituberculosis treatment in patients with difficult clinical conditions. Clin Respir J. (2015) 9:143–52. 10.1111/crj.12119 24521461

[8] Dartois VA Rubin EJ. Anti-tuberculosis treatment strategies and drug development: challenges and priorities. Nat Rev Microbiol. (2022) 20:685–701. 10.1038/s41579-022-00731-y 35478222 PMC9045034

[9] Rasouli MR Mirkoohi M Vaccaro AR Yarandi KK Rahimi-Movaghar V. Spinal tuberculosis: diagnosis and management. Asian Spine J. (2012) 6:294. 10.4184/asj.2012.6.4.294 23275816 PMC3530707

[10] Pandita A Madhuripan N Pandita S Hurtado RM. Challenges and controversies in the treatment of spinal tuberculosis. J Clin Tuberc Other Mycobact Dis. (2020) 19:100151. 10.1016/j.jctube.2020.100151 32154388 PMC7058908

[11] Alam MS Phan K Karim R Jonayed SA Munir HKM Chakraborty S Surgery for spinal tuberculosis: a multi-center experience of 582 cases. J Spine Surg. (2015) 1:65–71. 10.3978/j.issn.2414-469X.2015.07.03 27683681 PMC5039863

[12] Kilinc F Setzer M Behmanesh B Jussen D Geßler F Prinz V Surgical management and clinical outcome of cervical, thoracic and thoracolumbar spinal tuberculosis in a middle-European adult population. Sci Rep. (2023) 13:7000. 10.1038/s41598-023-34178-9 37117321 PMC10147912

[13] Kumar V Salaria AK Aggarwal A Dhatt SS. Surgical approaches in management of spinal tuberculosis. Ann Natl Acad Med Sci. (2021) 57:214. 10.1055/s-0041-1731596

[14] Tang Y Wu W Yang S Wang D-G Zhang Q Liu X Surgical treatment of thoracolumbar spinal tuberculosis—a multicentre, retrospective, case-control study. J Orthop Surg Res. (2019) 14:233. 10.1186/s13018-019-1252-4 31337417 PMC6651955

[15] Liu J Wan L Long X Huang S Dai M Liu Z. Efficacy and safety of posterior versus combined posterior and anterior approach for the treatment of spinal tuberculosis: a meta-analysis. World Neurosurg. (2015) 83:1157–65. 10.1016/j.wneu.2015.01.041 25698521

[16] Muheremu A Niu X Wu Z Tian W. Study on anterior and posterior approaches for spinal tuberculosis: a meta-analysis. Eur J Orthop Surg Traumatol. (2015) 25:69–76. 10.1007/s00590-014-1508-y 25047733

[17] Yang P Zang Q Kang J Li H He X. Comparison of clinical efficacy and safety among three surgical approaches for the treatment of spinal tuberculosis: a meta-analysis. Eur Spine J. (2016) 25:3862–74. 10.1007/s00586-016-4546-9 27029542

[18] Arifin J Biakto KT Johan MP Anwar StFZ. Clinical outcomes and surgical strategy for spine tuberculosis: a systematic review and meta-analysis. Spine Deform. (2023) 12(2):271–91. 10.1007/s43390-023-00785-9 37975989 PMC10867033

[19] Zhong Y Yang K Ye Y Huang W Liu W Luo J. Single posterior approach versus combined anterior and posterior approach in the treatment of spinal Tuberculosis: a meta-analysis. World Neurosurg. (2021) 147:115–24. 10.1016/j.wneu.2020.12.023 33316480

[20] Kelly A Younus A Lekgwara P. Minimally invasive spinal surgery in spinal tuberculosis—a case report series. Interdiscip Neurosurg. (2021) 23:101010. 10.1016/j.inat.2020.101010

[21] Lü G Wang B Li J Liu W Cheng I. Anterior debridement and reconstruction via thoracoscopy-assisted mini-open approach for the treatment of thoracic spinal tuberculosis: minimum 5-year follow-up. Eur Spine J. (2012) 21:463–9. 10.1007/s00586-011-2038-5 21997276 PMC3296839

[22] Kandwal P Garg B Upendra BN Chowdhury B Jayaswal A. Outcome of minimally invasive surgery in the management of tuberculous spondylitis. Indian J Orthop. (2012) 46:159–64. 10.4103/0019-5413.93680 22448053 PMC3308656

[23] Jayaswal A Upendra B Ahmed A Chowdhury B Kumar A. Video-assisted thoracoscopic anterior surgery for tuberculous spondylitis. Clin Orthop Relat Res. (2007) 460:100–7. 10.1097/BLO.0b013e318065b6e4 17471105

[24] Kelly A Younus A. Minimally invasive spinal surgery in spinal infections—a review. Interdiscip Neurosurg. (2020) 21:100749. 10.1016/j.inat.2020.100749

[25] Deng L Zhang Y-W Xiong L-Y Zhang S-L Ni W-Y Xiao Q. Extreme lateral interbody fusion and percutaneous pedicle screw fixation in the minimally invasive treatment of thoracic tuberculosis. J Int Med Res. (2020) 48:030006052092599. 10.1177/0300060520925992 PMC7278100 32459154

[26] Du X Ou Y Zhu Y Luo W Jiang G Jiang D. Oblique lateral interbody fusion combined percutaneous pedicle screw fixation in the surgical treatment of single-segment lumbar tuberculosis: a single-center retrospective comparative study. Int J Surg. (2020) 83:39–46. 10.1016/j.ijsu.2020.09.012 32927138

[27] Xu N-J Yu L Gu Y-J Wang X-Z Jiang W-Y Ma W-H. Minimally invasive direct lateral approach debridement, interbody bone grafting, and interbody fusion combined with posterior percutaneous pedicle screw fixation for lumbar spinal tuberculosis. Zhongguo Gu Shang. (2021) 34:228–34. 10.12200/j.issn.1003-0034.2021.03.008 33787166

[28] Xiong W Yu B Zhang Y Wang C Tang X Cao H Minimally invasive far lateral debridement combined with posterior instrumentation for thoracic and lumbar tuberculosis without severe kyphosis. J Orthop Surg Res. (2020) 15:221. 10.1186/s13018-020-01703-9 32546172 PMC7298961

[29] Donthu N Kumar S Mukherjee D Pandey N Lim WM. How to conduct a bibliometric analysis: an overview and guidelines. J Bus Res. (2021) 133:285–96. 10.1016/j.jbusres.2021.04.070

[30] Adnan S Lal A Naved N Umer F. A bibliometric analysis of scientific literature in digital dentistry from low- and lower-middle income countries. BDJ Open. (2024) 10:38. 10.1038/s41405-024-00225-4 38796474 PMC11127973

[31] İyibildiren M Eren T Ceran MB. Bibliometric analysis of publications on web of science database related to accounting information system with mapping technique. Cogent Bus Manag. (2023) 10. 10.1080/23311975.2022.2160584

[32] Manoj Kumar L George RJ Anisha PS. Bibliometric analysis for medical research. Indian J Psychol Med. (2023) 45:277–82. 10.1177/02537176221103617 37152388 PMC10159556

[33] Akhtar MN Haleem A Javaid M. Exploring the advent of medical 4.0: a bibliometric analysis systematic review and technology adoption insights. Inform Health. (2024) 1:16–28. 10.1016/j.infoh.2023.10.001

[34] Mirawati DK Wiyono N Ilyas MF Putra SE Hafizhan M. Research productivity in catamenial epilepsy: a bibliometric analysis of worldwide scientific literature (1956–2022). Heliyon. (2024) 10:e31474. 10.1016/j.heliyon.2024.e31474 38831810 PMC11145500

[35] Ilyas MF Lukas GA Lado A Rahmayani SA Tan K Benedictus B A bibliometric study of worldwide scientific literature on somatopsychics (1913‒2022). Bratisl Med J/Bratisl Lekarske Listy. (2024) 125:68. 10.4149/BLL_2024_68 38943506

[36] Dirgahayu P Ilyas MF Rahma AA Hanifa SN Wijayanto MA Triniputri WY Recent update on cerebral sparganosis: a bibliometric analysis and scientific mapping. Narra J. (2024) 4:e982. 10.52225/narra.v4i2.982 39280299 PMC11394178

[37] Dirgahayu P Wijayanto MA Triniputri WY Lukas GA Ilyas MF Rahma AA Cerebral toxoplasmosis in population with immunosuppressive therapy: a research trends analysis using bibliometrics and scientific mapping. J Med Pharm Chem Res. (2024) 7(3):431–46. 10.48309/jmpcr.2025.463783.1298

[38] Ilyas MF Lado A Indarta AF Madani BA Yarso KY Budhi IB. Worldwide research on abdominal compartment syndrome: bibliometric analysis of scientific literature (1993–2022). Gastroenterol Hepatol Bed Bench. (2024) 17(4). 10.22037/ghfbb.v17i4.2926 PMC12094512 40406437

[39] Wijayanto MA Lukas GA Greatalya LAD Ilyas MF Myrtha R. Global research trends and future direction in peripartum cardiomyopathy: a bibliometric analysis. Acta Inform Med. (2023) 31:270. 10.5455/aim.2023.31.270-274 38379692 PMC10875942

[40] Ghozali DA Doewes M Soetrisno S Indarto D Ilyas MF. A bibliometric analysis of 10 years of publications on L-citrulline. J Pharm Pharm Res. (2024) 12:2023. 10.56499/jppres23.1758_11.s1.27

[41] Sumarwoto T Ilyas MF Dewi A. Healthcare failure mode and effect analysis in surgery setting: a bibliometrics analysis and literature review. Acta Inform Med. (2023) 32:19–23. 10.5455/aim.2024.32.19-23 38585602 PMC10997166

[42] Cilmiaty R Ilyas MF. A bibliometrics and scientometrics study of mineral trioxide aggregate material for irreversible pulpitis. J Med Chem Sci. (2024) 7:729–43. 10.26655/JMCHEMSCI.2024.5.9

[43] Aria M. Cuccurullo *C. bibliometrix*: an R-tool for comprehensive science mapping analysis. J Informetr. (2017) 11:959–75. 10.1016/j.joi.2017.08.007

[44] Aria M Cuccurullo C D’Aniello L Misuraca M Spano M. Thematic analysis as a new culturomic tool: the social media coverage on COVID-19 pandemic in Italy. Sustainability. (2022) 14:3643. 10.3390/su14063643

[45] Aria M Misuraca M Spano M. Mapping the evolution of social research and data science on 30 years of social indicators research. Soc Indic Res. (2020) 149:803–31. 10.1007/s11205-020-02281-3

[46] van Eck NJ Waltman L. Citation-based clustering of publications using CitNetExplorer and VOSviewer. Scientometrics. (2017) 111:1053–70. 10.1007/s11192-017-2300-7 28490825 PMC5400793

[47] van Eck NJ Waltman L. Software survey: VOSviewer, a computer program for bibliometric mapping. Scientometrics. (2010) 84:523–38. 10.1007/s11192-009-0146-3 20585380 PMC2883932

[48] Garg RK Somvanshi DS. Spinal tuberculosis: a review. J Spinal Cord Med. (2011) 34:440–54. 10.1179/2045772311Y.0000000023 22118251 PMC3184481

[49] Jain AK. Tuberculosis of the spine. J Bone Joint Surg Br. (2010) 92-B:905–13. 10.1302/0301-620X.92B7.24668 20595106

[50] Hodgson AR Stock FE Fang HSY Ong GB. Anterior spinal fusion the operative approach and pathological findings in 412 patients with Pott’s disease of the spine. Br J Surg. (2005) 48:172–8. 10.1002/bjs.18004820819 13714863

[51] Moon M-S. Tuberculosis of the spine. Spine (Phila Pa 1976). (1997) 22:1791–7. 10.1097/00007632-199708010-00022 9259793

[52] Pertuiset E Beaudreuil J Lioté F Horusitzky A Kemiche F Richette P Spinal tuberculosis in adults: a study of 103 cases in a developed country, 1980–1994. Medicine (Baltimore). (1999) 78:309–20. 10.1097/00005792-199909000-00003 10499072

[53] Garg RK. Tuberculosis of the central nervous system. Postgrad Med J. (1999) 75:133–40. 10.1136/pgmj.75.881.133 10448488 PMC1741157

[54] Nussbaum ES Rockswold GL Bergman TA Erickson DL Seljeskog EL. Spinal tuberculosis: a diagnostic and management challenge. J Neurosurg. (1995) 83:243–7. 10.3171/jns.1995.83.2.0243 7616269

[55] Hodgson AR Stock FE. Anterior spinal fusion a preliminary communication on the radical treatment of Pott’s disease and Pott’s paraplegia. Br J Surg. (2005) 44:266–75. 10.1002/bjs.18004418508 13383153

[56] Oga M Arizono T Takasita M Sugioka Y. Evaluation of the risk of instrumentation as a foreign body in spinal tuberculosis. Spine (Phila Pa 1976). (1993) 18:1890–4. 10.1097/00007632-199310000-00028 8235878

[57] WHO. WHOGtcG (2015). Available online at: http://www.who.int/tb/publications/global_report/en/ (accessed September 5, 2024).

[58] Chen X Zhou J Yuan Q Zhang R Huang C Li Y. Challenge of ending TB in China: tuberculosis control in primary healthcare sectors under integrated TB control model–a systematic review and meta-analysis. BMC Public Health. (2024) 24:163. 10.1186/s12889-023-16292-5 38212753 PMC10785344

[59] Zhou J Yuan Q Huang Q Wang Q Huang H Chen W Implementation factors of tuberculosis control program in primary healthcare settings in China: a mixed-methods using the consolidated framework for implementation research framework. Infect Dis Poverty. (2024) 13:52. 10.1186/s40249-024-01222-3 38978081 PMC11229258

[60] Zhao W Zhu H. Extensive reforms to improve funding efficiency: an interview with president Xiankang dou of national natural science foundation of China. Natl Sci Rev. (2024) 11. 10.1093/nsr/nwae123 PMC11114466 38784101

[61] Liang X Zhang R Wang S Hang R Wang S Zhao Y Bibliometric analysis of medical and health research collaboration between China and ASEAN countries. Digit Health. (2023) 9. 10.1177/20552076231184993 PMC10327416 37426579

[62] Feng H Kurata K Cao J Itsuki K Niwa M Aoyama A Telemedicine research trends in 2001–2022 and research cooperation between China and other countries before and after the COVID-19 pandemic: bibliometric analysis. Interact J Med Res. (2024) 13:e40801. 10.2196/40801 39213010 PMC11399753

[63] Yuan J Wang S Pan C. Mechanism of impact of big data resources on medical collaborative networks from the perspective of transaction efficiency of medical services: survey study. J Med Internet Res. (2022) 24:e32776. 10.2196/32776 35318187 PMC9073602

[64] Punton J. Pott's disease. Its surgical treatment, with a report of a case.1. J Nerv Ment Dis. (1896) 21:770–5. 10.1097/00005053-189612000-00002

[65] Mandar B Menon VK Sameer P. Evolution of surgery for active spinal tuberculosis in adults: a narrative review. J Orthop Rep. (2024) 3:100312. 10.1016/j.jorep.2024.100312

[66] Zhu Z Hao D Wang B Gao W Yang R Guo H Selection of surgical treatment approaches for cervicothoracic spinal tuberculosis: a 10-year case review. PLoS One. (2018) 13:e0192581. 10.1371/journal.pone.0192581 29420648 PMC5805302

[67] Xue J Li T Song Y Liu H Liu L Gong Q. Treatment of severe bone destruction in L5-S1 spinal tuberculosis with anterior combined posterior approach. Research Square (2021). 10.21203/rs.3.rs-1111505/v134611661

[68] Liu R He J Fan Q Zhou H Wu X Yan Z Clinical efficacy of different surgical approaches in the treatment of thoracolumbar tuberculosis: a multicenter retrospective case–control study with a minimum 10-year follow-up. Int J Surg. (2024) 110(6):3178–89. 10.1097/JS9.0000000000001272 38498354 PMC11175723

[69] Karpushin AA Naumov DG Vishnevsky AA Nakaev AA. Thoracolumbar tuberculosis spondylitis: an analytical literature review of surgical reconstruction techniques. Genij Ortopedii. (2023) 29:104–9. 10.18019/1028-4427-2023-29-1-104-109

[70] He M Xu H Zhao J Wang Z. Anterior debridement, decompression, bone grafting, and instrumentation for lower cervical spine tuberculosis. Spine J. (2014) 14:619–27. 10.1016/j.spinee.2013.06.076 24314763

[71] Mohanty SP Pai Kanhangad M Yogesh Kumar B Singh A. Single-stage anterior debridement, posterior instrumentation and global fusion in thoracic and thoracolumbar tubercular spondylodiscitis. Musculoskelet Surg. (2019) 103:243–9. 10.1007/s12306-018-0581-5 30515742

[72] Kapoor SK Agarwal PN Jain BK Kumar R. Video-Assisted thoracoscopic decompression of tubercular spondylitis: clinical evaluation. Spine (Phila Pa 1976). (2005) 30:E605–10. 10.1097/01.brs.0000182328.03082.e2 16227877

[73] Garg N Vohra R. Minimally invasive surgical approaches in the management of tuberculosis of the thoracic and lumbar spine. Clin Orthop Relat Res. (2014) 472:1855–67. 10.1007/s11999-014-3472-6 24474323 PMC4016460

[74] Gan F Jiang J Xie Z Huang S Li Y Chen G Minimally invasive direct lateral interbody fusion in the treatment of the thoracic and lumbar spinal tuberculosis mini-DLIF for the thoracic and lumbar spinal tuberculosis. BMC Musculoskelet Disord. (2018) 19:283. 10.1186/s12891-018-2187-3 30086740 PMC6081909

[75] Yang X Luo C Liu L Song Y Li T Zhou Z Minimally invasive lateral lumbar intervertebral fusion versus traditional anterior approach for localized lumbar tuberculosis: a matched-pair case control study. Spine J. (2020) 20:426–34. 10.1016/j.spinee.2019.10.014 31669614

[76] Risantoso T Hidayat M Suyuti H Niam A. The role of instrumentation in the healing process of spinal tuberculosis: an experimental study. Open Access Maced J Med Sci. (2021) 9:457–63. 10.3889/oamjms.2021.6065

[77] Deng F Chen B Guo H Chen Q Wang F. Effectiveness and safety analysis of titanium mesh grafting versus bone grafting in the treatment of spinal tuberculosis: a systematic review and meta-analysis. BMC Surg. (2023) 23:377. 10.1186/s12893-023-02283-1 38087216 PMC10717474

[78] He Z Ou Y Hou B Wei J Mu X. A meta-analysis of the safety and effectiveness of titanium mesh versus bone graft alone for the treatment of thoracolumbar tuberculosis. Eur Spine J. (2020) 29:1505–17. 10.1007/s00586-019-06260-2 31872301

[79] Yin XH Liu ZK He BR Hao DJ. Single posterior surgical management for lumbosacral tuberculosis. Medicine (Baltimore). (2017) 96:e9449. 10.1097/MD.0000000000009449 29390579 PMC5758281

[80] Wu W Wang S Li Z Lin R Lin J. Posterior-only approach with titanium mesh cages versus autogenous iliac bone graft for thoracic and lumbar spinal tuberculosis. J Spinal Cord Med. (2021) 44:598–605. 10.1080/10790268.2019.1675953 31663833 PMC8288125

[81] Zhong W Liang X Tang K Luo X Quan Z. Transverse process strut and titanium mesh cages in the stability reconstruction of thoracic single segment tuberculosis: a retrospective single-center cohort study. BMC Musculoskelet Disord. (2020) 21:172. 10.1186/s12891-020-03196-3 32178643 PMC7077101

[82] Suya D Shao L Gu R Xu Q Luo W. Could nonstructural interbody fusion be an alternative surgical technique for treatment of single segment thoracic and lumbar spinal tuberculosis via a posterior- only approach? World Neurosurg. (2019) 130:e316–23. 10.1016/j.wneu.2019.06.072 31226454

[83] Gao Y Ou Y Deng Q He B Du X Li J. Comparison between titanium mesh and autogenous iliac bone graft to restore vertebral height through posterior approach for the treatment of thoracic and lumbar spinal tuberculosis. PLoS One. (2017) 12:e0175567. 10.1371/journal.pone.0175567 28407019 PMC5391077

[84] Koptan W ElMiligui Y ElSharkawi M. Single stage anterior reconstruction using titanium mesh cages in neglected kyphotic tuberculous spondylodiscitis of the cervical spine. Eur Spine J. (2011) 20:308–13. 10.1007/s00586-010-1537-0 20676701 PMC3030704

[85] Du X Ou Y Xu S He B Luo W Jiang D. Comparison of three different bone graft methods for single segment lumbar tuberculosis: a retrospective single-center cohort study. Int J Surg. (2020) 79:95–102. 10.1016/j.ijsu.2020.05.039 32442690

[86] Zhang H-Q Li M Wang Y-X Tang M-X Guo C-F Liu S-H Minimum 5-year follow-up outcomes for comparison between titanium mesh cage and allogeneic bone graft to reconstruct anterior column through posterior approach for the surgical treatment of thoracolumbar spinal tuberculosis with kyphosis. World Neurosurg. (2019) 127:e407–15. 10.1016/j.wneu.2019.03.139 30910755

[87] Li H Zhao C Huang X Hou T Zhang Z Xu J Application of autogenous iliac bone graft alone and iliac bone graft associated with titanium mesh on thoracolumbar spinal tuberculosis treatment. Chin J Front Med Sci (Electron Version). (2015) 7:120–4.

[88] Hu P Lu T. Comparison of the effects of autologous iliac bone and titanium mesh plus allogeneic bone fixation in the treatment of thoracic tuberculosis via transpedicular approach. Shandong Med J. (2017) 57:74–6.

[89] Li R-J Chen H Luo Y-J Lu Y-Y. A comparison between autologous bone graft and bone-filled titanium mesh cage graft in the treatment of lumbar spinal tuberculosis. J Shandong Univ (Health Sci). (2011) 49:115–8.

[90] Zhang Z Luo F Zhou Q Dai F Sun D Xu J. The outcomes of chemotherapy only treatment on mild spinal tuberculosis. J Orthop Surg Res. (2016) 11:49. 10.1186/s13018-016-0385-y 27177692 PMC4868010

[91] Aryal A Garg B Mehta N Shekhar S Gupta V. Is 6 months of antitubercular chemotherapy as effective as more than 6 months regimen in spinal tuberculosis? A systematic review and meta-analysis. Asian Spine J. (2022) 16:749–63. 10.31616/asj.2021.0104 34784702 PMC9633251

[92] Lin L Ke Z Cheng S. Efficacy and safety of short-term chemotherapy for patients with spinal tuberculosis undergoing surgery in Chinese population: a meta-analysis. J Orthop Surg Res. (2021) 16:229. 10.1186/s13018-021-02375-9 33781290 PMC8006363

[93] Party IC of MRMRCW. A controlled trial of short-course regimens of chemotherapy in patients receiving ambulatory treatment or undergoing radical surgery for tuberculosis of the spine. Indian J Tuberc. (1989) 36:1–21.

[94] Reetha AM Sivasubramanian S Parthasarathy R Somasundaram PR Prabhakar R. Five-year findings of a comparison of ambulatory short course chemotherapy with radical surgery plus chemotherapy for tuberculosis of the spine in madras. Indian J Orthop. (1994) 28:7–13.

[95] Medical Research Council Working Party on Tuberculosis of the Spine**.** Controlled trial of short-course regimens of chemotherapy in the ambulatory treatment of spinal tuberculosis. Results at three years of a study in Korea. Twelfth report of the medical research council working party on tuberculosis of the spine. J Bone Joint Surg Br. (1993) 75-B:240–8. 10.1302/0301-620X.75B2.8444944 8444944

[96] Darbyshire J. Five-year assessment of controlled trials of short-course chemotherapy regimens of 6, 9 or 18 months’ duration for spinal tuberculosis in patients ambulatory from the start or undergoing radical surgery. Int Orthop. (1999) 23:73–81. 10.1007/s002640050311 10422019 PMC3619789

[97] Banga RK Singh J Dahuja A Garg RS. Spinal tuberculosis—directly observed treatment and short course or daily anti tubercular therapy -are we over treating? Open Orthop J. (2018) 12:380–8. 10.2174/1874325001812010380 30369990 PMC6174613

[98] Medical Research Council Working Party on Tuberculosis of the Spine**.** A controlled trial of six-month and nine-month regimens of chemotherapy in patients undergoing radical surgery for tuberculosis of the spine in Hong Kong. Tubercle. (1986) 67:243–59. 10.1016/0041-3879(86)90014-0 2889281

[99] Nene AM Patil S Kathare AP Nagad P Nene A Kapadia F. Six versus 12 months of anti tubercular therapy in patients with biopsy proven spinal tuberculosis. Spine (Phila Pa 1976). (2019) 44:E1–6. 10.1097/BRS.0000000000002811 30045346

[100] Wang Z Shi J Geng G Qiu H. Ultra-short-course chemotherapy for spinal tuberculosis: five years of observation. Eur Spine J. (2013) 22:274–81. 10.1007/s00586-012-2536-0 23053764 PMC3555621

[101] Jiang J Gan F Tan H Xie Z Luo X Huang G Effect of computer navigation-assisted minimally invasive direct lateral interbody fusion in the treatment of patients with lumbar tuberculosis. Medicine (Baltimore). (2018) 97:e13484. 10.1097/MD.0000000000013484 30508977 PMC6283231

[102] Yasin P Yimit Y Cai X Aimaiti A Sheng W Mamat M Machine learning-enabled prediction of prolonged length of stay in hospital after surgery for tuberculosis spondylitis patients with unbalanced data: a novel approach using explainable artificial intelligence (XAI). Eur J Med Res. (2024) 29:383. 10.1186/s40001-024-01988-0 39054495 PMC11270948

[103] Duan S Dong W Hua Y Zheng Y Ren Z Cao G Accurate differentiation of spinal tuberculosis and spinal metastases using MR-based deep learning algorithms. Infect Drug Resist. (2023) 16:4325–34. 10.2147/IDR.S417663 37424672 PMC10329448

[104] Dong S Li W Tang Z-R Wang H Pei H Yuan B. Development and validation of a novel predictive model and web calculator for evaluating transfusion risk after spinal fusion for spinal tuberculosis: a retrospective cohort study. BMC Musculoskelet Disord. (2021) 22:825. 10.1186/s12891-021-04715-6 34563170 PMC8466716

[105] Hurley R. The BMJ awards India: and the winners are .. robotic spinal surgery, hospital stewardship of antibiotics, and primary care for hill tribes. Br Med J. (2014) 349:g5803. 10.1136/bmj.g5803 25253775

[106] Pan Q Yu H Li Y He X Shi J. Treatment of thoracolumbar tuberculosis with robot-assisted and minimally invasive access via transforaminal expansion approach. Zhongguo Xiu Fu Chong Jian Wai Ke Za Zhi. (2024) 38:935–41. 10.7507/1002-1892.202405079 39175314 PMC11335584

[107] Zhang T-T Wang Z-P Wang Z-H Wang Q-Y Xue W Song Y-X Accuracy and safety of robot assisted pedicle screw placement. Zhongguo Gu Shang. (2022) 35:108–12. 10.12200/j.issn.1003-0034.2022.02.003 35191259

[108] Bao B-X Yan H Tang J-G. Thoracic pedicle screw insertion assisted by the TiRobot system for spinal tuberculosis. Asian J Surg. (2021) 44:978–9. 10.1016/j.asjsur.2021.04.011 33947623

[109] Chhabra H Manghwani J. Robotic-assisted navigation guided kyphotic deformity correction surgery. Neurol India. (2022) 70:108. 10.4103/0028-3886.360927 36412355

[110] Fatima N Massaad E Hadzipasic M Shankar GM Shin JH. Safety and accuracy of robot-assisted placement of pedicle screws compared to conventional free-hand technique: a systematic review and meta-analysis. Spine J. (2021) 21:181–92. 10.1016/j.spinee.2020.09.007 32976997

[111] Wang L Li X Deng Z Cai Q Lei P Xu H Neuroendoscopic parafascicular evacuation of spontaneous intracerebral hemorrhage (NESICH technique): a multicenter technical experience with preliminary findings. Neurol Ther. (2024) 13:1259–71. 10.1007/s40120-024-00642-5 38914793 PMC11263518

[112] Zhang C Ge H Zhang S Liu D Jiang Z Lan C Hematoma evacuation via image-guided para-corticospinal tract approach in patients with spontaneous intracerebral hemorrhage. Neurol Ther. (2021) 10:1001–13. 10.1007/s40120-021-00279-8 34515953 PMC8571453

[113] Yu G Ding J Yang N Ge L Chen N Zhang X Evaluating the pro-survival potential of apoptotic bodies derived from 2D- and 3D- cultured adipose stem cells in ischaemic flaps. J Nanobiotechnology. (2024) 22:333. 10.1186/s12951-024-02533-1 38877492 PMC11177420

[114] Hao X Jiang B Wu J Xiang D Xiong Z Li C Nanomaterials for bone metastasis. J Contr Release. (2024) 373:640–51. 10.1016/j.jconrel.2024.07.067 39084467

